# Efficacy and Safety of Direct Oral Anticoagulants Versus Vitamin K Antagonists for Left Ventricular Thrombus: A Systematic Review and Meta-Analysis

**DOI:** 10.7759/cureus.84941

**Published:** 2025-05-28

**Authors:** Momina Khan, Kinnari Patel, Given E Edim, Maryam Mazhar, Hafiza Zanish Iram, Sandipkumar S Chaudhari, Calvin R Wei, Areeba Khan

**Affiliations:** 1 Acute Medicine, Heartlands Hospital, Birmingham, GBR; 2 Medicine, Sumy State University, La Mirada, USA; 3 School of Medicine, Raleigh General Hospital, Beckley, USA; 4 School of Medicine, Avalon University School of Medicine, Willemstad, CUW; 5 Internal Medicine, Services Hospital, Lahore, PAK; 6 Cardiothoracic Surgery, University of Alabama at Birmingham, Birmingham, USA; 7 Family Medicine, University of North Dakota School of Medicine and Health Sciences, Fargo, USA; 8 Research and Development, Shing Huei Group, Taipei, TWN; 9 Critical Care Medicine, United Medical and Dental College, Karachi, PAK

**Keywords:** anticoagulation therapy, direct oral anticoagulants, left ventricular thrombus, thromboembolism, vitamin k antagonists

## Abstract

Left ventricular thrombus (LVT) is a serious complication associated with myocardial infarction and cardiomyopathy. While vitamin K antagonists (VKAs) have been the standard treatment, direct oral anticoagulants (DOACs) offer potential advantages. This meta-analysis compares the efficacy and safety of DOACs versus VKAs in patients with LVT. We conducted a systematic search of PubMed, Excerpta Medica database (Embase), Scopus, Web of Science, and Cochrane Central Register of Controlled Trials (CENTRAL) from inception to April 2025. Studies comparing DOACs with VKAs in adult patients with LVT were included. The primary outcome was LVT resolution; secondary outcomes included stroke or systemic embolism (SSE) and bleeding events. Random-effects models were used to calculate pooled risk ratios (RRs) with 95% confidence intervals. Thirty-five studies (five RCTs and 30 observational) with 4,823 patients were included. LVT resolution was comparable between DOACs and VKAs (RR: 1.04, 95% CI: 0.99-1.10, I² = 30%). Risk of SSE showed no significant difference (RR: 0.91, 95% CI: 0.80-1.03, I² = 11%). DOACs were associated with significantly lower bleeding risk (RR: 0.87, 95% CI: 0.81-0.94, I² = 0%). Subgroup analyses by study design showed consistent findings. DOACs demonstrated comparable efficacy to VKAs for LVT resolution and stroke prevention while offering a superior safety profile with lower bleeding risk. These findings suggest DOACs may be a viable alternative to VKAs in LVT management, though large-scale randomized trials are needed to confirm these results and establish optimal dosing strategies.

## Introduction and background

Left ventricular thrombus (LVT) is a serious and potentially life-threatening complication commonly associated with conditions such as anterior myocardial infarction, heart failure with reduced ejection fraction, and non-ischemic cardiomyopathy [1,2]. The formation of a thrombus in the left ventricle predisposes patients to systemic embolization, including stroke, and therefore mandates timely and effective anticoagulation therapy [3]. Traditionally, vitamin K antagonists (VKAs), such as warfarin, have been the cornerstone of treatment for LVT due to their established efficacy in thrombus resolution and prevention of thromboembolic events [4]. However, VKAs require regular monitoring, have a narrow therapeutic index, and are associated with multiple drug and dietary interactions, which limit their utility in real-world clinical practice [5]. 

Anticoagulation is critical in preventing devastating complications of LVT, particularly ischemic stroke and peripheral embolism [2]. Recent interest has focused on direct oral anticoagulants (DOACs) as potential alternatives to VKAs, given their predictable pharmacokinetics, fixed dosing, and fewer interactions [4]. Despite these advantages, the evidence supporting DOAC use in LVT remains limited and evolving. Current guidelines, such as those from the American College of Cardiology and the European Society of Cardiology, continue to recommend VKAs as the standard of care, highlighting the need for robust comparative data to inform clinical decision-making [6].

In recent years, DOACs, including agents such as apixaban, rivaroxaban, dabigatran, and edoxaban, have emerged as alternatives to VKAs in various thromboembolic conditions, including atrial fibrillation and venous thromboembolism [6]. DOACs offer several advantages over VKAs, including fixed dosing, fewer drug-food interactions, and no need for routine coagulation monitoring. These benefits have led to growing interest in their potential role in the management of LVT [7]. Despite the increasing off-label use of DOACs for LVT, current international guidelines remain cautious, citing limited high-quality evidence to support their routine use in this indication. 

VKAs exert their anticoagulant effect by inhibiting the enzyme vitamin K epoxide reductase, which is essential for the post-translational carboxylation of clotting factors II, VII, IX, and X [8]. This inhibition reduces the synthesis of these vitamin K-dependent coagulation factors, thereby impairing thrombin generation and clot formation [8]. In contrast, DOACs provide targeted inhibition of key components in the coagulation cascade. Dabigatran directly inhibits thrombin (factor IIa), while apixaban, rivaroxaban, and edoxaban selectively inhibit activated factor X (Xa) [9]. By directly targeting specific coagulation factors, DOACs offer a more predictable pharmacological profile, fewer interactions, and rapid onset of action, eliminating the need for routine coagulation monitoring [10]. These pharmacologic differences have raised the question of whether DOACs can serve as a reliable alternative to VKAs for indications such as LVT.

To date, few comprehensive meta-analyses have assessed the comparative effectiveness and safety of DOACs versus VKAs specifically for LVT [11,12]. New studies have been conducted since then. Given the clinical importance of preventing systemic embolism while minimizing bleeding complications, a better understanding of the relative benefits and risks of these agents in this population is urgently needed through analyzing recent evidence. Therefore, the aim of this systematic review and meta-analysis is to evaluate and compare the efficacy of DOACs and VKAs in achieving LVT resolution, as well as their impact on stroke prevention and bleeding events.

## Review

Methodology 

Literature Search and Search Strategy 

A comprehensive literature search was conducted to identify all relevant studies comparing direct oral anticoagulants (DOACs) with vitamin K antagonists (VKAs) in the treatment of left ventricular thrombus (LVT). We systematically searched PubMed, Excerpta Medica database (Embase), Scopus, Web of Science, and the Cochrane Central Register of Controlled Trials (CENTRAL) from inception to 10 April 2025. The search strategy included combinations of keywords and Medical Subject Headings (MeSH) related to “left ventricular thrombus,” “LVT,” “direct oral anticoagulants,” “DOACs,” “non-vitamin K oral anticoagulants,” “NOACs,” “apixaban,” “rivaroxaban,” “dabigatran,” “edoxaban,” and “warfarin.” Boolean operators (AND, OR) were used to optimize the search sensitivity. Reference lists of relevant reviews and eligible studies were also manually screened for additional studies. All search and screening steps were independently performed by two reviewers. Discrepancies were resolved through discussion or in consultation with a third reviewer. 

Study Selection 

All identified citations were imported into EndNote (Clarivate Plc, PA, USA) for deduplication. Two reviewers independently screened titles and abstracts for eligibility. Full-text articles of potentially relevant studies were then assessed to determine inclusion. Studies were included if they enrolled adult patients (≥18 years) with a diagnosis of LVT, compared DOACs with VKAs, and reported at least one of the following outcomes: LVT resolution, stroke, or bleeding events. Both randomized controlled trials (RCTs) and observational studies were considered. Exclusion criteria included case reports, reviews, editorials, conference abstracts without full data, and studies lacking comparative outcome data. Any disagreement between the reviewers regarding eligibility was resolved by consensus or discussion with a third reviewer.

Data Extraction and Outcomes 

Two reviewers independently extracted data using a standardized data collection form. Extracted information included study characteristics (author, publication year, country, study design), patient demographics (sample size, mean age, proportion of females), clinical features (presence of diabetes, hypertension), details of anticoagulant treatment, and length of follow-up. Outcomes of interest included LVT resolution (primary outcome), stroke, and bleeding events (secondary outcomes), as defined by each study. Both adjusted and unadjusted effect estimates (RRs, ORs, HRs) along with 95% confidence intervals (CIs) were extracted. Where effect estimates were not reported, they were calculated from raw data when available. Disagreements in data extraction were resolved by discussion between the reviewers or by involving a third reviewer.

Statistical Analysis 

Statistical analyses were performed using random-effects models (DerSimonian and Laird method) to account for potential heterogeneity among studies. Pooled risk ratios (RRs) with 95% CIs were calculated for dichotomous outcomes. A p-value less than 0.05 was considered significant. Heterogeneity was assessed using the I² statistic, with thresholds of 25%, 50%, and 75% representing low, moderate, and high heterogeneity, respectively. Subgroup analyses based on study design (randomized controlled trial vs. observational) were conducted to assess the consistency of effect estimates. All statistical analyses were conducted using Review Manager (RevMan version 5.4.1, The Cochrane Collaboration, London, UK).

Results 

**Figure 1:**
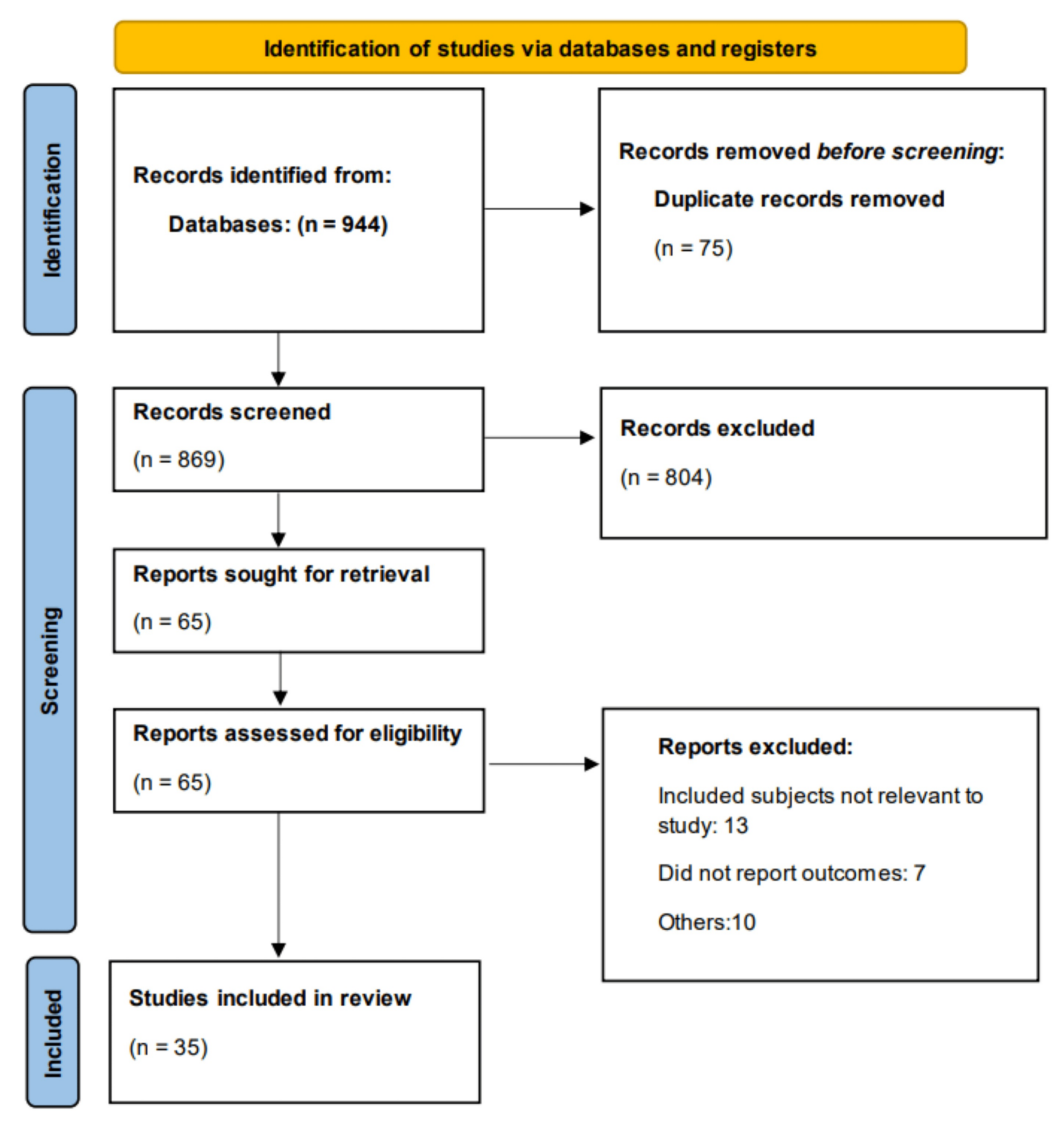
PRISMA flowchart PRISMA: Preferred Reporting Items for Systematic Reviews and Meta-Analyses

Figure 1 shows the Preferred Reporting Items for Systematic Reviews and Meta-Analyses (PRISMA) flowchart of study selection. Overall, 944 studies were identified through online database searching. After removing 75 duplicates, the remaining records were initially screened. The full text of 65 studies was obtained, and a detailed assessment was done. Finally, 35 studies were included in the meta-analysis. Table 1 presents characteristics of the included studies. Out of all included studies, five were RCTs and 30 were observational (one prospective observational and 29 retrospective observational), with sample sizes ranging from 18 to 949. Follow-up duration of included studies ranged from three to 64.8 months. The included studies were published between 2018 and 2025. A total of 35 studies were conducted across diverse geographic regions. The majority originated from the United States (14 studies), followed by the United Kingdom (five), China (four), Saudi Arabia (two), Portugal (two), Malaysia (two), and one study each from Iran, Israel, Kenya, Switzerland, France, and various African nations. Table 2 presents demographic characteristics of participants enrolled in the included studies. The mean or median age across studies was generally comparable between groups, ranging from the early 50s to mid-60s in both arms. Female representation varied but was generally lower. Among studies reporting comorbidities, diabetes and hypertension were frequently documented. Diabetes prevalence in the DOAC arms ranged from one to 45 individuals, while in the warfarin arms it ranged from one to 124 individuals. Similarly, hypertension was common across both groups, with up to 90 patients in the DOAC arms and up to 217 in the warfarin arms being affected. However, several studies had missing or unreported data for one or more baseline characteristics. Despite variability, the demographic and clinical profiles between DOAC and warfarin users appeared broadly similar across the studies. Table 3 presents the quality assessment of included studies.

**Table 1:** Characteristics of the included studies RCT: randomized controlled trial; NR: not reported; DOAC: direct oral anticoagulant; VKA: vitamin K agonist

Author	Year	Region	Study Design	Groups	Sample Size	Type of DOAC	Follow-up Duration
Abdelnabi et al. [13]	2021	Africa	RCT	DOAC	39	Rivaroxaban	6 Months
				VKA	40		
Al-Abcha et al. [14]	2025	United States	Retrospective Observational	DOAC	46	Apixaban, rivaroxaban, dabigatran, or edoxaban	NR
				VKA	143		
Albabtain et al. [15]	2021	Saudi Arabia	Retrospective Observational	DOAC	28	Rivaroxaban	9.5 Months
				VKA	35		
Alcalai et al. [16]	2022	Israel	RCT	DOAC	18	Apixaban	3 Months
				VKA	17		
Aldaas et al. [17]	2022	United States	Retrospective Observational	DOAC	75	NR	10.0 Months
				VKA	142		
Alizadeh et al. [18]	2019	United Kingdom	Prospective Observational	DOAC	38	Apixaban, rivaroxaban, or edoxaban	21.6 Months
				VKA	60		
Ali et al. [19]	2020	United States	Retrospective Observational	DOAC	32	Apixaban, rivaroxaban and dabogatran	12 Months
				VKA	60		
Bass et al. [20]	2021	United States	Retrospective Observational	DOAC	180	Apixaban, rivaroxaban and dabogatran	3 Months
				VKA	769		
Cochran et al. [21]	2021	United States	Retrospective Observational	DOAC	14	Apixaban, rivaroxaban, dabigatran, or edoxaban	12 Months
				VKA	59		
Conant et al. [22]	2022	United States	Retrospective Observational	DOAC	29	NR	NR
				VKA	135		
Daher et al. [23]	2020	France	Retrospective Observational	DOAC	17	Apixaban, rivaroxaban and dabogatran	64.8 Months
				VKA	42		
Gama et al. [24]	2019	Portugal	Retrospective Observational	DOAC	12	NR	6 Months
				VKA	52		
Guddeti et al. [25]	2020	United States	Retrospective Observational	DOAC	19	Apixaban, rivaroxaban and dabogatran	10.4 Months
				VKA	80		
Herald et al. [26]	2022	United States	Retrospective Observational	DOAC	134	Apixaban, rivaroxaban, dabigatran, or edoxaban	40.8 Months
				VKA	299		
Iqbal et al. [27]	2020	United Kingdom	Retrospective Observational	DOAC	22	NR	36 Months
				VKA	62		
Isa et al. [28]	2020	Malaysia	RCT	DOAC	14	Apixaban	3 Months
				VKA	13		
Jaidka et al. [29]	2018	United Kingdom	Retrospective Observational	DOAC	12	Apixaban, rivaroxaban and dabogatran	6 Months
				VKA	37		
Jenab et al. [30]	2025	Iran	RCT	DOAC	26	Rivaroxaban	3 Months
				VKA	24		
Jones et al. [31]	2021	United Kingdom	Retrospective Observational	DOAC	41	Apixaban, rivaroxaban, or edoxaban	26.4 Months
				VKA	60		
Lim et al. [32]	2019	Malaysia	Retrospective Observational	DOAC	5	NR	24 Months
				VKA	18		
Mihim et al. [33]	2021	United States	Retrospective Observational	DOAC	33	Apixaban, rivaroxaban, dabigatran, or edoxaban	6 Months
				VKA	75		
Minciunescu et al. [34]	2020	United States	Retrospective Observational	DOAC	57	NR	NR
				VKA	140		
Paiva et al. [35]	2025	Portugal	Retrospective Observational	DOAC	99	Apixaban, rivaroxaban, dabigatran, or edoxaban	24 Months
				VKA	72		
Rahunathan et al. [36]	2023	United Kingdom	Retrospective Observational	DOAC	14	NR	4.66 Months
				VKA	4		
Robinson et al. [37]	2020	United States	Retrospective Observational	DOAC	121	Apixaban	11.7 Months
				VKA	236		
Seiler et al. [38]	2023	Switzerland	Retrospective Observational	DOAC	48	Apixaban and Rivaroxaban	31.3 Months
				VKA	53		
Tamimi et al. [39]	2022	United States	Retrospective Observational	DOAC	48	Apixaban, rivaroxaban, or edoxaban	6 Months
				VKA	116		
Varwani et al. [40]	2021	Kenya	Retrospective Observational	DOAC	36	Apixaban, rivaroxaban and dabogatran	12 Months
				VKA	25		
Willeford et al. [41]	2021	United States	Retrospective Observational	DOAC	22	Apixaban, rivaroxaban, dabigatran, or edoxaban	8.5 Months
				VKA	129		
Xu et al. [42]	2021	China	Retrospective Observational	DOAC	25	Dabigatran and rivaroxaban	28.44 Months
				VKA	62		
Yang et al. [43]	2023	China	Retrospective Observational	DOAC	91	NR	3 Months
				VKA	33		
Yao et al. [44]	2025	United Kingdom	Retrospective Observational	DOAC	19885	Apixaban, rivaroxaban, dabigatran, or edoxaban	3 Months
				VKA	19885		
Youssef et al. [45]	2023	Saudi Arabia	RCT	DOAC	25	Apixaban	6 Months
				VKA	25		
Yunis et al. [46]	2020	United States	Retrospective Observational	DOAC	64	NR	24 Months
				VKA	200		
Zhang et al. [47]	2022	China	Retrospective Observational	DOAC	33	Rivaroxaban	8.5 Months
				VKA	31		
Zhou et al. [48]	2024	China	Retrospective Observational	DOAC	111	Apixaban, rivaroxaban and dabogatran	6 Months
				VKA	129		

**Table 2:** Characteristics of participants enrolled in each study DOAC: direct oral anticoagulant; VKA: vitamin K agonist; NR: not reported

Author ID	Groups	Total Population	Mean Age (Years)	Female (n)	Diabetes (n)	Hypertension (n)
Abdelnabi et al., 2021 [13]	DOAC	39	NR	NR	NR	NR
	VKA	40				
Al-Abcha et al., 2025 [14]	DOAC	46	62.8	9	15	31
	VKA	143	60.8	38	49	100
Albabtain et al., 2021 [15]	DOAC	28	58.25	4	12	13
	VKA	35	59	1	16	19
Alcalai et al., 2022 [16]	DOAC	18	55.2	5	8	7
	VKA	17	58.8	2	5	7
Aldaas et al., 2022 [17]	DOAC	75	NR	NR	NR	NR
	VKA	142				
Alizadeh et al., 2019 [18]	DOAC	38	NR	NR	NR	NR
	VKA	60				
Ali et al., 2020 [19]	DOAC	32	59.2	6	12	NR
	VKA	60	58	11	18	
Bass et al., 2021 [20]	DOAC	180	63.4	55	NR	NR
	VKA	769	61.6	224		
Cochran et al., 2021 [21]	DOAC	15	51.5	3	7	NR
	VKA	59	62	14	23	
Conant et al., 2022 [22]	DOAC	29	NR	NR	NR	NR
	VKA	135				
Daher et al., 2020 [23]	DOAC	17	57	3	2	10
	VKA	42	61	7	9	17
Gama et al., 2019 [24]	DOAC	12	NR	NR	NR	NR
	VKA	52				
Guddeti et al., 2020 [25]	DOAC	19	60.7	4	NR	NR
	VKA	80	61.3	25		
Herald et al., 2022 [26]	DOAC	134	66	18	45	90
	VKA	299	65	57	124	217
Iqbal et al., 2020 [27]	DOAC	22	62	2	19	9
	VKA	62	62	7	19	18
Isa et al., 2020 [28]	DOAC	14	55.36	1	7	8
	VKA	13	55	1	9	9
Jaidka et al., 2018 [29]	DOAC	12	57.2	3	1	2
	VKA	37	61.3	9	7	18
Jenab et al., 2025 [30]	DOAC	26	55	4	7	9
	VKA	24	55	5	5	14
Jones et al., 2021 [31]	DOAC	41	58.73	8	7	23
	VKA	60	60.81	9	10	22
Lim et al., 2019 [32]	DOAC	5	NR	NR	NR	NR
	VKA	18				
Mihim et al., 2021 [33]	DOAC	33	63.3	10	8	24
	VKA	75	60.3	21	20	56
Minciunescu et al., 2020 [34]	DOAC	57	60.4	11	15	46
	VKA	140	59.5	34	39	97
Paiva et al., 2025 [35]	DOAC	99	55.4	13	20	47
	VKA	72	65.9	17	10	67
Rahunathan et al., 2023 [36]	DOAC	14	58.5	2	3	4
	VKA	4	63.5	1	1	0
Robinson et al., 2020 [37]	DOAC	121	58.1	27	36	86
	VKA	236	58.2	66	92	177
Seiler et al., 2023 [38]	DOAC	48	64.3	6	8	24
	VKA	53	62.2	12	11	31
Tamimi et al., 2022 [39]	DOAC	48	NR	NR	NR	NR
	VKA	116				
Varwani et al., 2021 [40]	DOAC	36	NR	NR	NR	NR
	VKA	25				
Willeford et al., 2021 [41]	DOAC	22	54	5	4	8
	VKA	129	56	25	37	54
Xu et al., 2021 [42]	DOAC	25	59.4	6	6	10
	VKA	62	61.9	15	12	27
Yang et al., 2023 [43]	DOAC	91	NR	NR	NR	NR
	VKA	33				
Yao et al., 2025 [44]	DOAC	19885	61.8	6320	7575	14983
	VKA	19885	61.6	6310	7560	14997
Youssef et al., 2023 [45]	DOAC	25	52	NR	12	11
	VKA	25	53		11	10
Yunis et al., 2020 [46]	DOAC	64	NR	NR	NR	NR
	VKA	200				
Zhang et al., 2022 [47]	DOAC	33	60.3	9	10	23
	VKA	31	61.3	8	5	11
Zhou et al., 2024 [48]	DOAC	111	56	19	41	48
	VKA	129	55	10	46	63

**Table 3:** Quality assessment of included observational studies (Newcastle-Ottawa Scale)

Author ID	Selection	Comparison	Assessment	Overall Grade
Al-Abcha et al., 2025 [14]	4	1	3	Good
Albabtain et al., 2021 [15]	4	1	2	Good
Aldaas et al., 2022 [17]	3	0	2	Fair
Alizadeh et al., 2019 [18]	3	0	3	Fair
Ali et al., 2020 [19]	3	0	2	Good
Bass et al., 2021 [20]	3	1	2	Fair
Cochran et al., 2021 [21]	3	1	3	Good
Conant et al., 2022 [22]	3	2	2	Good
Daher et al., 2020 [23]	3	1	3	Good
Gama et al., 2019 [24]	3	1	2	Good
Guddeti et al., 2020 [25]	3	2	3	Good
Herald et al., 2022 [26]	3	1	3	Good
Iqbal et al., 2020 [27]	3	2	3	Good
Jaidka et al., 2018 [29]	3	0	3	Fair
Jones et al., 2021 [31]	3	1	3	Good
Lim et al., 2019 [32]	3	0	2	Good
Mihim et al., 2021 [33]	3	1	3	Good
Minciunescu et al., 2020 [34]	3	1	2	Fair
Paiva et al., 2025 [35]	4	2	3	Good
Rahunathan et al., 2023 [36]	3	1	3	Good
Robinson et al., 2020 [37]	3	1	3	Good
Seiler et al., 2023 [38]	4	1	3	Good
Tamimi et al., 2022 [39]	2	0	2	Poor
Varwani et al., 2021 [40]	3	1	3	Good
Willeford et al., 2021 [41]	3	1	3	Good
Xu et al., 2021 [42]	3	0	3	Fair
Yang et al., 2023 [43]	4	1	3	Good
Yao et al., 2025 [44]	4	1	3	Good
Yunis et al., 2020 [46]	3	0	2	Fair
Zhang et al., 2022 [47]	3	1	3	Good
Zhou et al., 2024 [48]	3	1	2	Fair

Meta-analysis of Outcomes 

**Figure 2:**
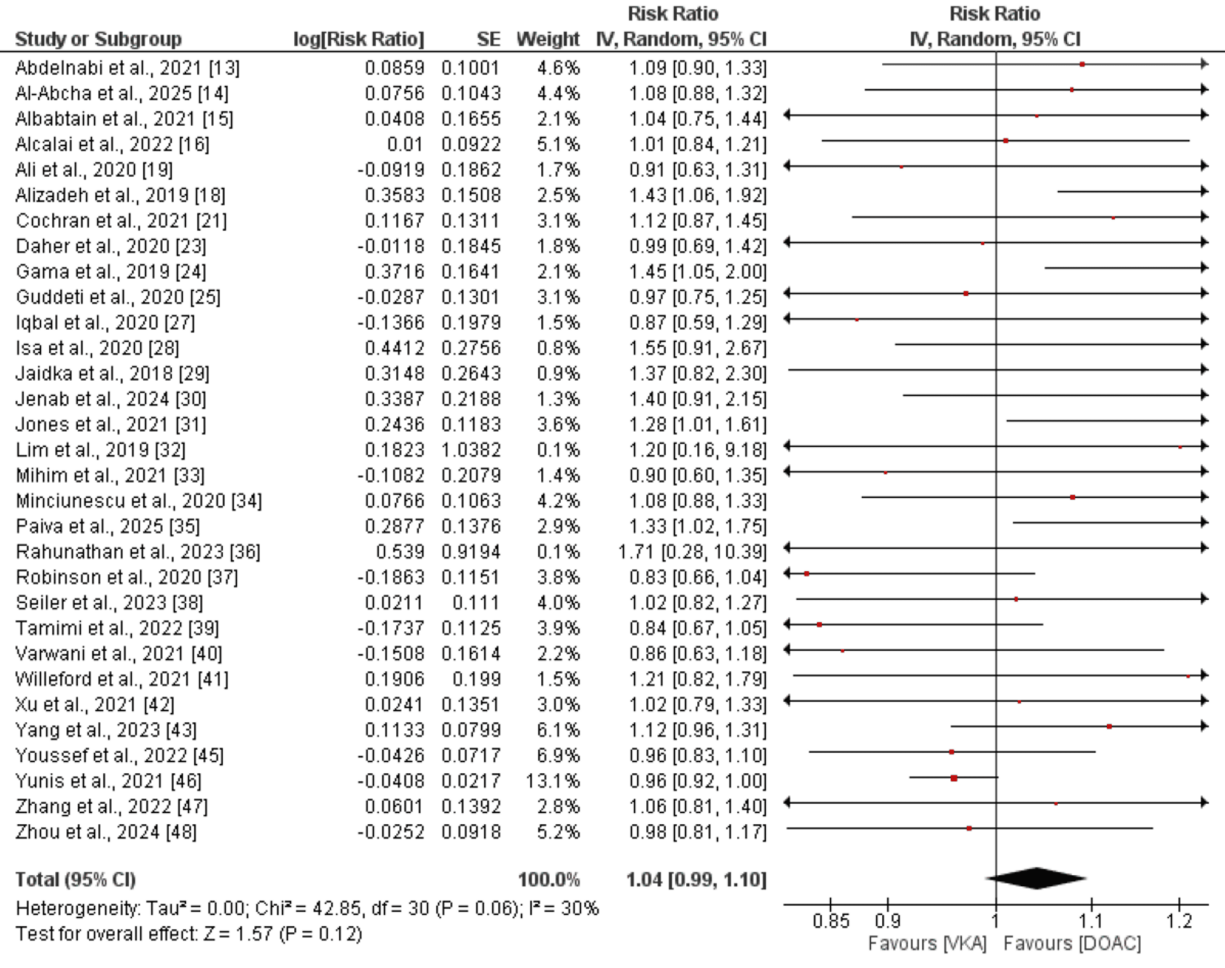
Comparison of thrombus resolution between DOAC and LVT DOAC: direct oral anticoagulant; LVT: left ventricular thrombus [13-16, 18,19, 21, 23-25, 27-43, 45-48]

LVT resolution: Comparison of LVT resolution between DOAC and VKA was done by performing a pooled analysis of 31 studies, and the results are presented in Figure 2. Pooled analysis showed that LVT resolution was not significantly different in subjects receiving DOAC compared to VKA (RR: 1.04, 95% CI: 0.99 to 1.10). Moderate heterogeneity was reported among the study results (I-Square: 30%). 

**Figure 3:**
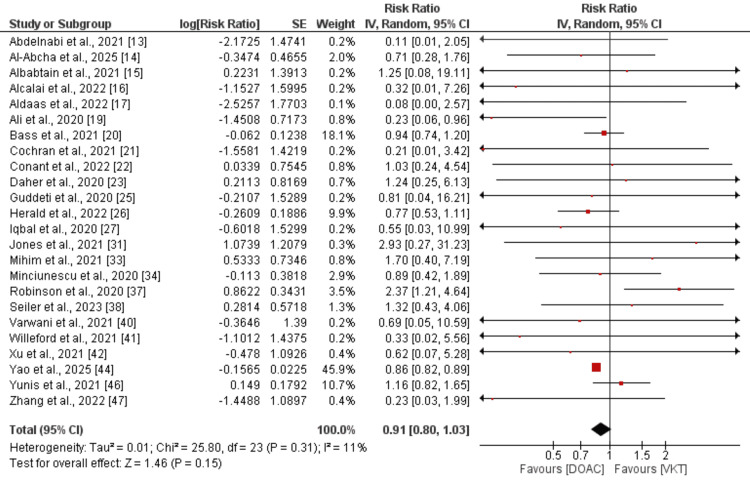
Comparison of the risk of SSE between DOAC and VKT [13-17, 19-23, 25-27, 31, 33,34, 37,38, 40-42, 44, 46,47] SSE: systemic embolism; DOAC: direct oral anticoagulant; VKT: vitamin K antagonist

Stroke or systemic embolism (SSE): Comparison of SSE between DOAC and VKA was done by performing a pooled analysis of 24 studies, and the results are presented in Figure 3. Pooled analysis showed that the risk of SSE was not significantly different between subjects receiving DOAC compared to VKA (RR: 0.91, 95% CI: 0.80 to 1.03). Low heterogeneity was reported among the study results (I-Square: 11%). As shown in the pooled analysis, most of the weight was carried by one study, i.e., Yao et al. [44]. We performed a sensitivity analysis by removing that study. Pooled effect still showed no significant difference is there between the two groups in terms of risk of SSE (RR: 0.95, 95% CI: 0.79 to 1.15). 

**Figure 4:**
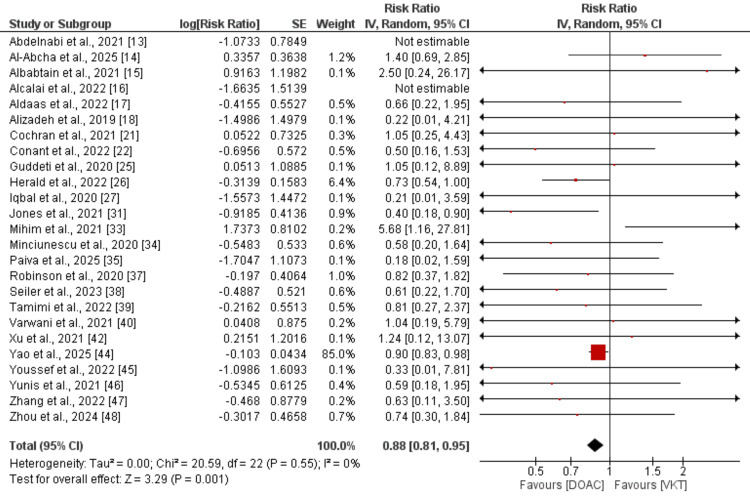
Comparison of the risk of bleeding events between DOAC and VKA [13-18, 21-22, 25-27, 31, 33-35, 37-40, 42, 44-48] DOAC: direct oral anticoagulants; VKA: vitamin K antagonist

Bleeding events: Comparison of the risk of stroke between DOAC and VKA was done by performing a pooled analysis of 25 studies, and the results are presented in Figure 4. Pooled analysis showed that the risk of bleeding events was lower in subjects receiving DOAC compared to VKA, and this difference was statistically significant (RR: 0.88, 95% CI: 0.81 to 0.95). No heterogeneity was reported among the study results (I-Square: 0%). As shown in the pooled analysis, most of the weight was carried by one study, i.e., Yao et al. [44]. We performed a sensitivity analysis by removing that study. Pooled effect still showed low risk of bleeding event in DOAC group (RR: 0.73, 95% CI: 0.60 to 0.89). 

Subgroup Analysis 

Subgroup analyses based on study design (RCTs vs. observational studies) were conducted for the outcomes of LVT resolution, stroke, and bleeding events, and the results are presented in Table 4. For LVT resolution, both RCTs and observational studies demonstrated no significant difference between DOACs and VKAs. The pooled RR from RCTs was 1.05 (95% CI: 0.93 to 1.19; I² = 19%), and from observational studies was 1.04 (95% CI: 0.98 to 1.10; I² = 22%), indicating consistent findings with low heterogeneity across both study designs.

**Table 4:** Subgroup analysis based on study design RR: risk ratio; CI: confidence interval; RCT: randomized controlled trial; LVT: left ventricular thrombus

Outcomes	Subgroups	RR (95% CI)	I-Square
LVT Resoultion	RCT	1.05 (0.93 to 1.19)	19%
	Observational	1.04 (0.98 to 1.10)	22%
Stroke	RCT	0.18 (0.02 to 1.52)	0%
	Observational	0.91 (0.80 to 1.03)	6%
Bleeding Events	RCT	0.31 (0.09 to 1.07)	0%
	Observational	0.88 (0.81 to 0.95)	0%

In terms of stroke prevention, DOACs showed a potentially protective effect in RCTs, with an RR of 0.18 (95% CI: 0.02 to 1.52; I² = 0%), although the wide CI suggests imprecision due to a small sample size. Observational studies showed a modest trend favoring DOACs, with an RR of 0.91 (95% CI: 0.80 to 1.03; I² = 6%), though the effect was not statistically significant. Regarding bleeding events, DOACs were associated with a lower risk compared to VKAs. In RCTs, the pooled RR was 0.31 (95% CI: 0.09 to 1.07; I² = 0%), suggesting a trend toward reduced bleeding with DOACs, albeit not statistically significant. Observational studies provided stronger evidence, showing a significant reduction in bleeding risk with an RR of 0.88 (95% CI: 0.81 to 0.95; I² = 0%).

These subgroup findings support the overall trend that DOACs may offer similar efficacy to VKAs for LVT resolution and stroke prevention, while potentially conferring a lower risk of bleeding, especially in real-world observational settings. 

Discussion

This systematic review and meta-analysis aimed to compare the efficacy and safety of DOACs versus VKAs in patients with LVT. Our findings suggest that DOACs are comparable to VKAs in terms of efficacy for LVT resolution and stroke prevention. Furthermore, DOACs demonstrated a favorable safety profile, with a lower risk of bleeding events compared to VKAs.

Multiple prior meta-analyses have similarly demonstrated that there is no statistically significant difference in LVT resolution rates between DOACs and VKAs. For example, a meta-analysis by Li et al. [49], which included 17 studies, reported an RR of 1.07 for LVT resolution with DOACs compared to VKAs (95% CI: 0.97-1.18; p = 0.193). In another meta-analysis encompassing 21 studies, Huang et al. [50] observed a comparable effect size, with an RR of 1.06 (95% CI: 0.98-1.13; p = 0.13), reinforcing the conclusion that both anticoagulant strategies yield similar outcomes in promoting LVT resolution. Our current meta-analysis updates these findings by including recently conducted RCTs and observational studies.

The most effective anticoagulation strategy for managing LVT remains uncertain. Existing clinical guidelines continue to advocate for the use of warfarin as the standard treatment in this setting. Despite its proven efficacy, warfarin presents several drawbacks that limit its practicality, such as the need for regular INR monitoring, significant interactions with food and other medications, a prolonged half-life, and challenges with maintaining patient adherence due to its narrow therapeutic range [51].

Our meta-analysis demonstrated that the rate of LVT resolution is similar between patients receiving DOACs and those treated with VKAs. Achieving thrombus resolution is a critical therapeutic goal, as the presence of LVT is associated with a substantially worse prognosis, including an increased risk of major adverse cardiovascular events, serious bleeding, overall mortality, and a higher likelihood of embolic events [52].

The optimal length of anticoagulant therapy in patients with LVT has yet to be clearly defined. Evidence suggests that the risk of thromboembolic events is particularly elevated during the initial two weeks following a myocardial infarction, while the likelihood of LVT recurrence peaks within the first three months post-infarction [53]. Recent guidance from the American Heart Association recommends follow-up imaging around the three-month mark after acute myocardial infarction, with the discontinuation of anticoagulation if no thrombus is observed [3]. In individuals with dilated cardiomyopathy complicated by LVT, a minimum treatment duration of three to six months is typically advised [54].

In conclusion, this meta-analysis provides evidence supporting the use of DOACs as a treatment option for LVT. Our findings suggest that DOACs offer similar efficacy to VKAs while potentially providing a better safety profile with reduced bleeding risk. Nonetheless, confirmation through large-scale randomized controlled trials with sufficient sample sizes and extended follow-up periods remains necessary.

This meta-analysis has several important limitations that temper the confidence in recommending DOACs as a first-line alternative for LVT management. Firstly, most included studies were retrospective, with variable follow-up durations and clinical settings, introducing potential bias and limiting causal inference. Considerable heterogeneity was observed across studies in terms of patient populations, antithrombotic regimens, and imaging modalities, complicating direct comparisons and the generalizability of results. Most studies relied on echocardiography for assessing LVT resolution, which may overestimate thrombus clearance compared to more sensitive imaging methods like cardiac MRI. There was also substantial variability in antiplatelet therapy use, and due to insufficient data, we could not assess outcomes of anticoagulation alone versus combination therapy with antiplatelets. Similarly, the optimal DOAC dosing strategies and treatment durations remain unclear, as these were inconsistently reported and not uniformly evaluated across studies. Subgroup analyses based on clinical scenarios such as acute coronary syndrome (ACS) or percutaneous coronary intervention (PCI) were not feasible due to the lack of detailed stratified data. These gaps highlight the need for further investigation in specific patient subgroups, standardized imaging methods, and clearer documentation of adjunctive therapies. Although some findings appeared consistent across RCTs and observational studies, this consistency varied by outcome and study design. For example, the observed bleeding risks were more pronounced in observational data, suggesting potential differences in patient selection or unmeasured confounding.

Overall, while the results provide valuable insights, they must be interpreted cautiously. Future large-scale, high-quality RCTs are essential to definitively establish the comparative efficacy and safety of DOACs versus VKAs in LVT treatment. These should also explore tailored treatment approaches based on patient characteristics, DOAC dosing regimens, and concurrent antiplatelet use to inform optimal clinical decision-making.

## Conclusions

This comprehensive meta-analysis of 35 studies comparing DOACs to VKAs in patients with LVT demonstrates that DOACs provide comparable efficacy in thrombus resolution and stroke prevention while offering a superior safety profile with significantly reduced bleeding risk. These findings challenge the current paradigm of VKAs as the exclusive standard of care for LVT management. The consistent results across randomized controlled trials and observational studies strengthen the validity of our conclusions. However, limitations in study design, follow-up duration, and imaging techniques necessitate cautious interpretation. Further large-scale randomized controlled trials with extended follow-up periods are essential to definitively establish DOACs as a first-line alternative to VKAs for LVT management and to determine optimal dosing regimens and treatment durations.
